# Preventing tibial and talar component contact during implantation of a total ankle replacement

**DOI:** 10.1308/003588413X13511609957056c

**Published:** 2013-01

**Authors:** AJ Roche, JD Calder

**Affiliations:** Chelsea and Westminster Hospital NHS Foundation Trust, UK

## Background

The MOBILITY™ ankle replacement (DePuy, Leeds, UK) is the most frequently used implant in the UK.^1^ After bony preparation of articulating surfaces, the arthroplasty implants must be manoeuvred carefully into definitive position while preventing inadvertent scratching of bearing surfaces that could lead to wear and early failure.^2,3^ We describe an easy way of protecting the talar component while implanting the tibial component.

## Technique

**Figure 1:**
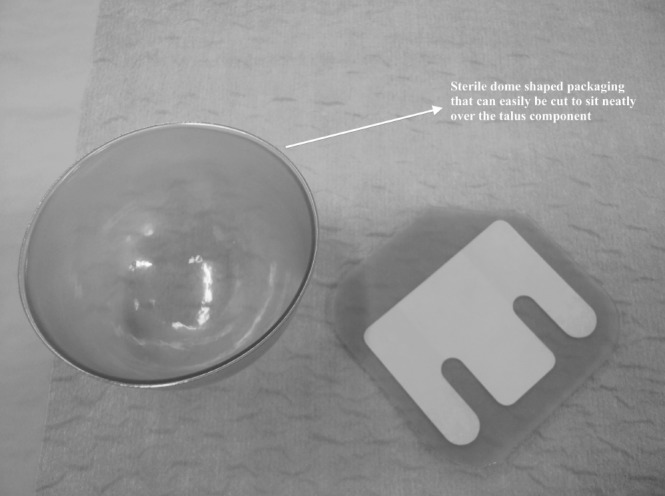
The sterile packaging accompanying the components can be safely cut to size and used to cover the talus component.

**Figure 2:**
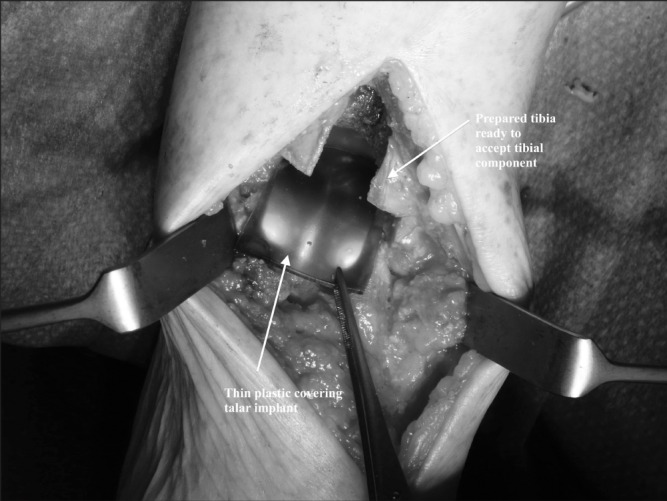
The cut plastic packaging is a perfect contour to fit neatly over the talus component without risking abrasive damage to the tibia.

We use the anterior approach to the ankle.^4^ Once the correct components are chosen following trial insertions, they are removed from their sterile packaging. The talus is implanted first as per the manufacturer’s technique. We use the thin plastic covering accompanying the sterile tibial implant (Fig 1) to protect the talus during tibial insertion. The dome-shaped plastic covering can be trimmed to a smaller talar-sized piece to cover the talus, leaving an unobstructed view during tibial component insertion in the standard fashion (Fig 2). With both implants seated, the plastic can be exchanged for the implant bearing.

## Discussion

The trial bearing can be used to protect the talus, as suggested by the manufacturer. However, this could dislodge or partially obstruct tibial component insertion, potentially increasing the risk of contact between metal bearings. The plastic covering we use is partially transparent, which can assist the surgeon in viewing the talus and determine whether the talus component displaces during tibial implantation. This is a simple and safe method to carefully insert the tibial component of a commonly used ankle replacement.
